# Simultaneous mapping of EMCD signals and crystal orientations in a transmission electron microscope

**DOI:** 10.1038/s41598-021-81071-4

**Published:** 2021-01-26

**Authors:** Hasan Ali, Jan Rusz, Tobias Warnatz, Björgvin Hjörvarsson, Klaus Leifer

**Affiliations:** 1 Applied Materials Science, Department of Materials Science and Engineering, Uppsala University, Box 534, 75121 Uppsala, Sweden; 2 Department of Physics and Astronomy, Uppsala University, Box 516, 75120 Uppsala, Sweden; 3 Department of Electrical Engineering, Mirpur University of Science and Technology (MUST), Mirpur, 10250 AJK Pakistan; 4 Present Address: Department of Materials and Environmental Chemistry, Stockholm University, 10691 Stockholm, Sweden

**Keywords:** Ferromagnetism, Magnetic properties and materials, Characterization and analytical techniques, Scanning electron microscopy, Transmission electron microscopy

## Abstract

When magnetic properties are analysed in a transmission electron microscope using the technique of electron magnetic circular dichroism (EMCD), one of the critical parameters is the sample orientation. Since small orientation changes can have a strong impact on the measurement of the EMCD signal and such measurements need two separate measurements of conjugate EELS spectra, it is experimentally non-trivial to measure the EMCD signal as a function of sample orientation. Here, we have developed a methodology to simultaneously map the quantitative EMCD signals and the local orientation of the crystal. We analyse, both experimentally and by simulations, how the measured magnetic signals evolve with a change in the crystal tilt. Based on this analysis, we establish an accurate relationship between the crystal orientations and the EMCD signals. Our results demonstrate that a small variation in crystal tilt can significantly alter the strength of the EMCD signal. From an optimisation of the crystal orientation, we obtain quantitative EMCD measurements.

## Introduction

Electron magnetic circular dichroism (EMCD)^1^, a transmission electron microscope (TEM) based technique, has emerged as an important technique to determine the magnetic moments of the materials with much higher spatial resolution as compared to its X-ray counterpart XMCD^2^. The EMCD technique was proposed in 2003^3^ and experimentally demonstrated in 2006^4^. From the time of its discovery, EMCD has seen a continuous improvement in the signal to noise (S/N) ratio^5–10^, the spatial resolution^11–16^ and the quantitative analysis. The technique has also been applied to explore material based questions such as interfacial magnetism^17,18^, magnetocrystalline anisotropy^19^, properties of dilute magnetic semiconductors^20^ and rare earth magnets^21^. Recently EMCD signals with single atomic plane resolution were achieved^22,23^.

One of the apparently simple findings in the experimental evolution of the EMCD technique since its discovery is that the sample orientation and the electron beam position must be very well defined in order to obtain a quantitative EMCD signal. The accurate knowledge of sample orientation is thus of uttermost importance when aiming for quantitative atomic resolution EMCD. With this in mind, it is suprising that hitherto, the orientation dependence of the EMCD signal has not been analysed systematically in the experimental situation. Furthermore, when aiming for atomically resolved EMCD work, not only the orientation must be precisely known, but also the position of the electron beam with respect to the exposed atom must be well defined and strictly identical for both EELS spectra that are needed for obtaining the EMCD signal. Having acquired the STEM image of the atomic lattice of the magnetic material, the place of the EMCD analysis can be accurately determined. But, it is non-trivial to obtain all information needed for highly accurate EMCD, i.e. orientation and both EELS spectra simultaneously.

In the classical EMCD experimental setup, the TEM sample is tilted to a 2-beam condition (2BC) and two electron energy loss (EELS) spectra are acquired at two conjugate scattering angles. From an experimental point of view, tilting the TEM sample to a perfect 2BC is not always trivial especially for thin samples and nanoparticles where the Kikuchi lines are not visible. Depending on the thickness and the extinction length of the material, the intensity of the diffracted beam can be lower or even higher than the direct beam. Another difficulty arises when acquiring the spatial maps of EMCD in scanning TEM (STEM) mode. Even if the magnetic materials are nearly perfect single crystals, the crystal orientation of the TEM sample might locally change within the measured area, thus producing different orientation conditions at different scan points. In fact, most magnetic materials have small misorientations between different regions of the sample resulting in texture angles of the order of a few mrad so the precise orientation alignment remains a challenge. It has been shown in simulations that a deviation from a 2BC can produce a change in the strength and distribution of the EMCD signals in the reciprocal space^24^ but no systematic experimental study has been carried out in this context. One of the major difficulties in such work consists in the serial acquisition of the two EELS spectra needed for the EMCD and the diffraction patterns at each beam position which make it hard to ensure the spatial registration among these measurements.

Here, we have developed a technique to simultaneously map both conjugate EELS spectra and the local orientation of the sample in a single acquisition and thus can obtain all signals, EMCD signal and sample orientation simultaneously at each point of a scanned map. This enables the determination of the effect of crystal tilt on the measured EMCD signal with high accuracy. By inserting a custom-made quadruple aperture (QA)^25^ in the reciprocal space plane of the electron beam trajectory, we simultaneously obtain four angle-resolved spectroscopic signals in a single acquisition. The four signals include the two conjugate EELS spectra required for the EMCD measurements and the inelastic intensities of the **0** and the **g** beams (in 2-beam orientation). The approach is based on the hypothesis that a ratio of the inelastic intensities of the diffracted (${I}_{g}^{^{\prime}}$) and the direct (${I}_{0}^{^{\prime}}$) beams quantitatively represent the local orientation of the crystal. In fact, we show by our simulations how the values of ${I}_{g}^{^{\prime}}/{I}_{0}^{^{\prime}}$ are related to a tilt of the crystal from exact 2-beam orientation. We establish an experimental relationship between the crystal orientation and the EMCD signals and demonstrate that a change in crystal orientation significantly affects the measured magnetic signals. We find the exact tilt direction in the experiments by analyzing the elastic intensities ${I}_{0}$ and ${I}_{g}$ of the **0** and the **g** beams respectively by acquiring elastic diffraction patterns immediately after the QA-mapping.

## Results

### Simulations

To verify the hypothesis that the values of ${I}_{g}^{^{\prime}}/{I}_{0}^{^{\prime}}$ at each beam position are representative of the specimen’s orientation we have performed simulations of diffraction patterns for both elastic and inelastic electron scattering of a parallel beam on an iron sample tilted into a systematic row orientation, while varying the Laue circle center.

Computational details were set in the following way. Beam acceleration voltage was set to 300 kV, as in the experiment. The sample was tilted by approximately 10 degrees from the (001) zone axis into the systematic row orientation. This brings the sample into the three-beam orientation with the same strength of excitation of both – **g** and + **g** = (200) beams. On top of that we introduced a tilt parallel to the systematic row, varying within a range from − 6 mrad to + 20 mrad with a step of 1 mrad, i.e., in total we have calculated diffraction patterns for 27 different beam orientations. Sample thicknesses up to approximately 30 nm were considered, but in the figures below we focus on the thickness range from 15 to 25 nm, centred on the experimental sample thickness of 20 nm. Calculations were performed using MATS.v2 code^26^, modified to also output the relevant information from the elastic scattering of the incoming beam. We have calculated diffraction patterns for inelastic scattering of electrons within the Fe L_3_-edge energy range and *θ*_x_ = (− 2 mrad, 16 mrad) and *θ*_y_ = (− 12 mrad, 12 mrad) ranges of the scattering angles. MATS.v2 convergence parameter was set to 2 × 10^–5^, well sufficient for a plane-wave calculation.

**Figure 1:**
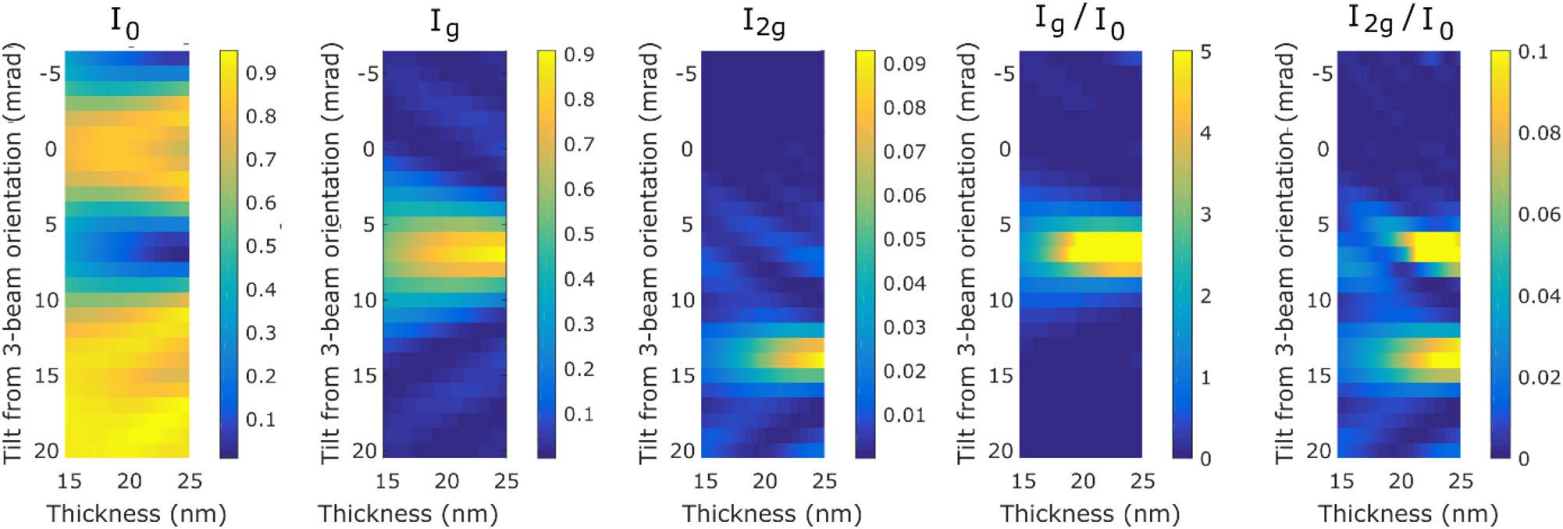
Results from elastic scattering calculations of the intensities of the transmitted beam and the Bragg scattered beams **g** = (200) and 2**g** as a function of tilt from three-beam orientation and sample thickness for bcc iron crystal at 300 kV acceleration voltage.

First we summarize results of the calculations of the elastic scattering. In the experiments, a diffraction mapping using elastically scattered electrons was performed over approximately the same area on which the map containing the spectroscopic information was taken. From this dataset, the intensities of ${I}_{0}$, ${I}_{g}$ and ${I}_{2g}$ were extracted. Figure 1 shows the elastic scattering calculation results of intensities of these beams and their ratios as a function of sample thickness and the tilt from the 3-beam orientation. The most prominent feature is the strong reduction of the intensity of the transmitted beam at around 7 mrad beam tilt, especially at a thickness approaching 25 nm. This tilt is close to the exact 2-beam orientation i.e. where the excitation error of the **g** beam is close to zero. (Note a similar feature developing towards a beam tilt of − 7 mrad, when it would be the -**g** beam approaching low excitation error.) Simultaneously, the intensity of the **g** beam increases. This is an example of well-known Pendellösung oscillations. At approximately 14 mrad tilt, the 2**g** beam approaches zero excitation error and its intensity grows in that region. However, this beam has a significantly higher extinction distance and therefore at thicknesses below 25 nm it does not develop significant intensity. The ratio of the **g**-beam and the transmitted beam intensities highlights the intensity oscillation even more clearly, reaching very high values near the exact two-beam orientation, especially for higher sample thicknesses where the sample thickness gets closer to the extinction length. The ratio ${I}_{2g}/{I}_{0}$ shows two local maxima, one near the exact two-beam orientation, which is due to the afore-mentioned minimum of the transmitted beam intensity, and the second near the tilt that minimizes the excitation error of the 2**g** beam—bringing thus its intensity up.

**Figure 2:**
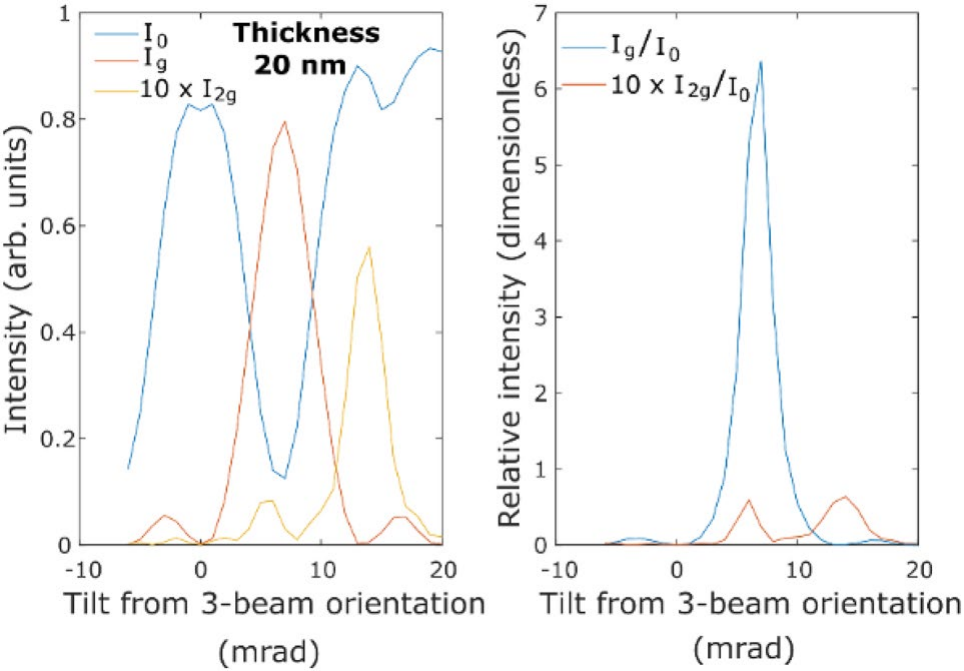
Intensities of transmitted beam and Bragg-scattered beams **g** and **2g** and their ratios as a function of beam tilt for a 20 nm thick sample of bcc iron.

A more detailed insight, particularly relevant for the interpretation of experiments conducted in this work, is provided by the results at sample thickness of 20 nm. Figure 2 shows line profiles with beam intensities and their ratios as a function of the tilt. The intensity of **g** beam ${I}_{g}$ is well above the intensity of the transmitted beam ${I}_{0}$, when the sample is close to an exact two-beam orientation. The intensity ratio ${I}_{g}/{I}_{0}$ peaks at a value of more than 6. On the other hand the 2**g** beam (due to its large extinction distance) has very low intensities across the whole range of the tilts (note that its intensity was scaled up by factor of 10 for better visibility in the Fig. 2). At any orientation, its relative intensity does not go above approximately 5–6% of the transmitted beam intensity at this sample thickness.

**Figure 3:**
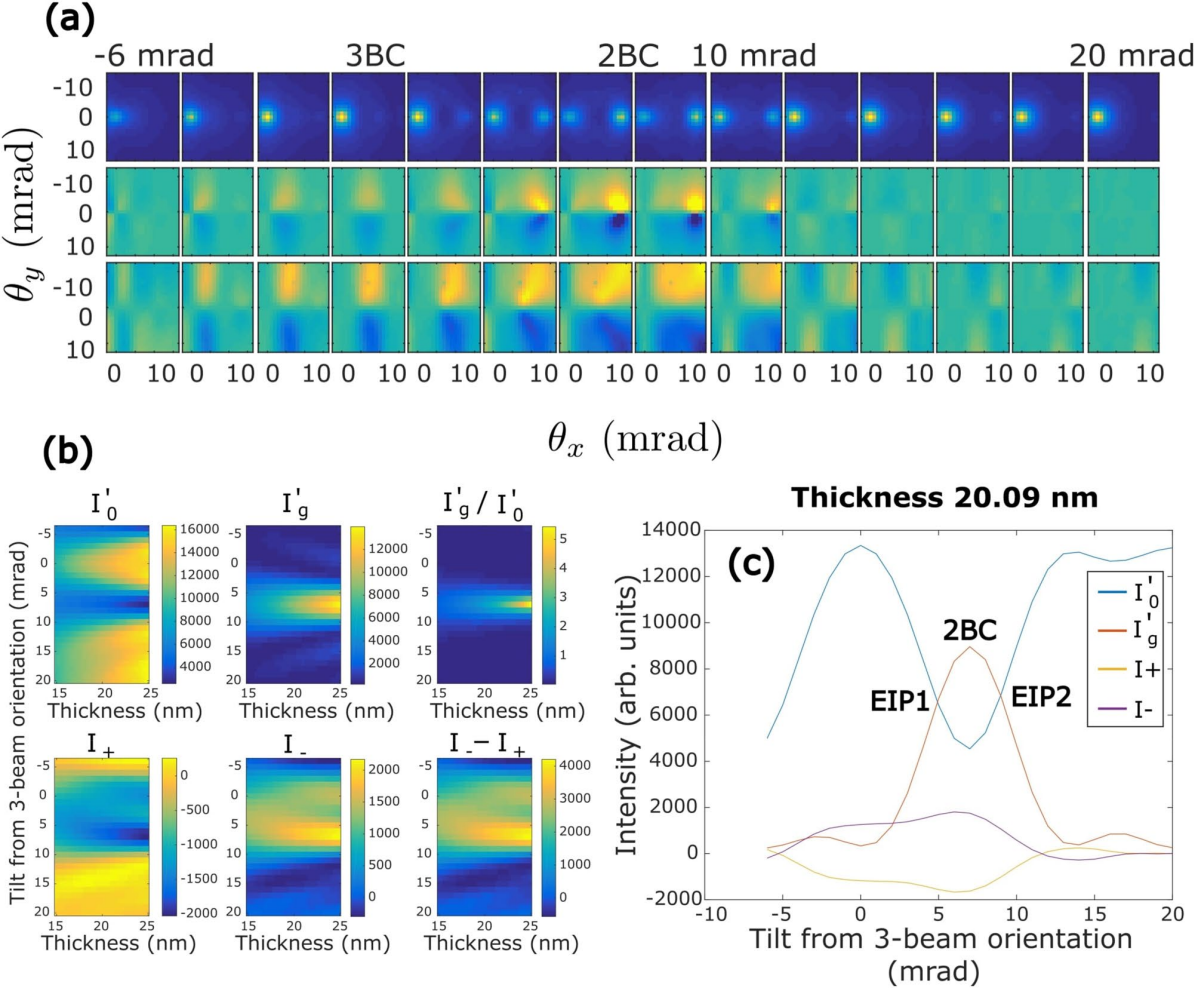
**(a)** Non-magnetic (top row), magnetic (EMCD; middle row) and relative magnetic (bottom row) diffraction patterns at Fe L_3_ edge for calculated tilts from the three-beam orientation at sample thickness of 20 nm** (b)** inelastic intensities of **0** and **g** beam and their ratio as a function of sample thickness and tilt from 3BC **(c)** inelastic intensities of **0** and **g** beam and the magnetic signals plotted as a function of tilt from 3-beam orientation.

We proceed with the results of inelastic scattering calculations. Figure 3a shows the non-magnetic (top row), magnetic (EMCD; middle row) and relative magnetic (bottom row) diffraction patterns at Fe L_3_ edge for all calculated tilts from the three-beam orientation at sample thickness of 20 nm. Note, how the area with the strongest magnetic signal moves throughout the diffraction plane. In absolute strength it is largest in the vicinity of the **g** beam, although there it is superposed on tails of the large non-magnetic signal. For that reason, the relative strength of the magnetic signal is not necessarily optimal there. Overall, the relative strength of the magnetic signal seems to be rather stable near the Thales circle detector positions, when the beam tilt is near the two-beam orientation.

These diffraction patterns were further analysed by placing virtual detector apertures as in the experiment. That effectively reduces the diffraction patterns down to four number per beam tilt, representing the non-magnetic intensities of inelastically scattered electrons at the transmitted beam and **g** beam and the magnetic intensities at **( +)** and **(−)** detector positions. Figure 3b summarizes these results as a function of tilt and sample thickness, including the ratio of intensities at the transmitted beam and the **g** beam. Qualitatively the results for non-magnetic components of the inelastic scattering cross-section strongly remind the results of the elastic calculations shown in Fig. 1. This is due to preservation of the elastic contrast, as discussed elsewhere^27–30^.

Reducing the data further, we focus in Fig. 3c on the four inelastic intensities at a sample thickness of 20 nm. Again, the non-magnetic results are qualitatively similar to the results from elastic calculations shown in Fig. 2, however there are quantitative differences. For example, the **g** beam intensity only reaches about 60% of the maximal intensity of the transmitted beam, while in the elastic calculation it was up to about 90%. These quantitative differences might be related to the limited small detector aperture used to collect the inelastic scattering intensities (only 1 mrad semi-angle). However, we have not explored this effect further.

In view of the experimental results on EMCD, shown below, we note that in both the elastic and inelastic calculations, equal intensities of the transmitted beam and the **g**-beam are not obtained at the exact 2-beam orientation for the sample thickness of 20 nm. Instead, the equal intensities are obtained at two different tilts, at approximately 2–2.5 mrad away from the exact two beam orientation. For simplicity, we will refer these two points as ‘equal intensity points (EIP)’ in the coming text. In addition, we point out that the EMCD strength (both absolute and relative) is not maximal, when the intensities of the transmitted and **g** beam are equal.

## Experimental results

**Figure 4:**
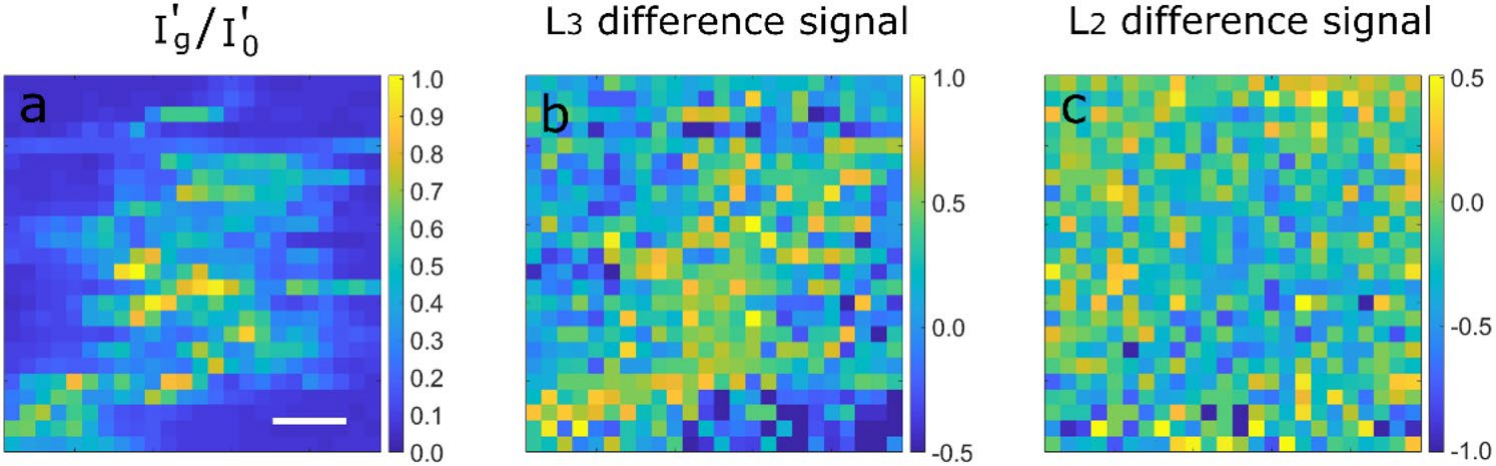
The maps showing the values of **(a)** I’_g_/I’_0_ **(b)** L_3_ difference and **(c)** L_2_ difference in real space. A clear correlation can be seen among the maps where the areas with higher I’_g_/I’_0_ values result in positive difference at L_3_ and negative difference at L_2_. The scale bar is 20 nm.

To verify the simulations shown in Fig. 3, we acquired 4D STEM dataset from a 100 × 100 nm^2^ area of the Fe sample (for details, see “Methods” section). We calculated the ratio of inelastic intensities of the **0** and the **g** beams (${I}_{g}^{^{\prime}}/{I}_{0}^{^{\prime}}$) and the difference signals from the L_3_ and L_2_ energy loss edges of Fe from the spectroscopic images at each scan point of the 4D dataset (Methods Section). The results are presented in Fig. 4 where the three maps represent the same area of the sample which was scanned by the electron beam. The ${I}_{g}^{^{\prime}}/{I}_{0}^{^{\prime}}$ map shows a region of similar orientation within the measured area which is oriented close to 2-beam condition (2BC) whereas the orientation of the regions surrounding the well-oriented grain is quite far away from the 2BC. A clear correlation can be observed among the three maps. The well-oriented region of the sample results in large positive values of L_3_ difference signal and negative values of L_2_ difference signal as expected by the EMCD signature. The L_3_ and L_2_ difference signals, on the other hand, weaken or even change their sign in the misoriented regions. Thus it seems that a change in crystal orientation away from the 2BC significantly influences the measured EMCD signals. We have also seen such evolution in Fig. 3a where a crystal tilt from 2BC results in substantial variations in the EMCD signal strength and of its distribution within the diffraction plane.

**Figure 5:**
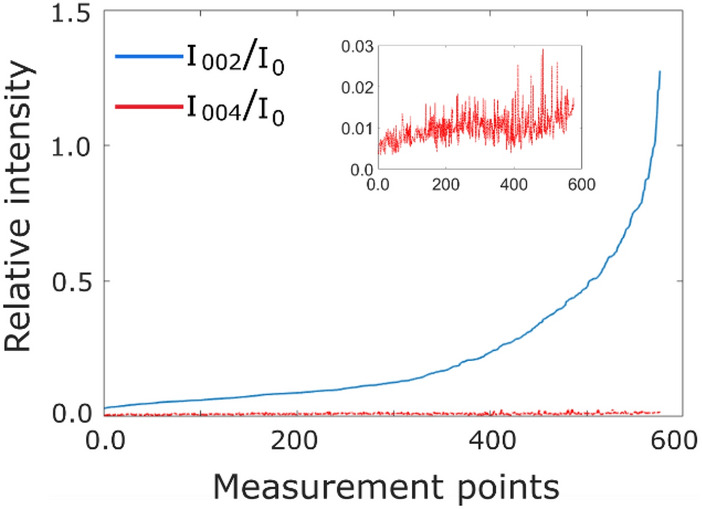
The quantities I_002_/I_0_ and I_004_/I_0_ are plotted together in an order from the lowest to the highest values of I_002_/I_0_. The intensities are extracted from a 4D diffraction data cube.

It is important to note that we obtain the maximum value of ${I}_{g}^{^{\prime}}/{I}_{0}^{^{\prime}}$ = 1 in the experimental data. Thus in these maps, the orientation closest to the 2BC is 2–2.5 mrad off which corresponds to the sample orientation denoted by the equal intensity points (EIPs) in Fig. 3c. The interval of crystal orientations in the analysed area of the sample can then be either from EIP1 towards 3-beam orientation or from EIP2 towards away from the 3-beam orientation. Having determined the crystal orientation experimentally allows for a comparison of the data to the simulations. To find the interval of crystal orientations in our experiment, we acquired a 4D diffraction scan dataset from nearly the same region measured in the above experiment. This dataset contains an elastic diffraction pattern at each scan point. This dataset was acquired immediately after the QA-scan and in the already set conditions the 0-, **g** and 2**g** (004 for Fe) beam was captured in the diffraction patterns. The intensity changes in **g** and 2**g** beams can be used to find out the tilt interval of the sample in the QA-scan. We calculated the elastic intensity ratios of ${I}_{g}/{I}_{0}$ and ${I}_{2g}/{I}_{0}$ from the 4D diffraction dataset. The intensity ratios were plotted in the following way. First, we order and plot the ${I}_{g}/{I}_{0}$ intensities in the diffraction dataset from lowest to highest intensity, then we plot ${I}_{2g}/{I}_{0}$ intensity taken from the same measurement point of the map at the same number along x-axis defined by ${I}_{g}/{I}_{0}$ as shown in Fig. 5. According to the simulations shown in Fig. 2, considering a tilt direction from EIP2 towards away from the 3-beam orientation, this would cause an increase in ${I}_{2g}/{I}_{0}$ with a decrease in ${I}_{g}/{I}_{0}$. On the other hand, if we consider the tilt direction from EIP1 towards 3-beam orientation, ${I}_{2g}/{I}_{0}$ does not show any significant change and stays at very low values with a decrease in ${I}_{g}/{I}_{0}$. The experimental intensity plots presented in Fig. 5 clearly match the latter case confirming that the equal intensities of ${I}_{0}$ and ${I}_{g}$ obtained in our experiments represent EIP1in the simulations and the interval of sample tilt for the points in the map ranges from the 2BC towards the 3-beam orientation.

**Figure 6:**
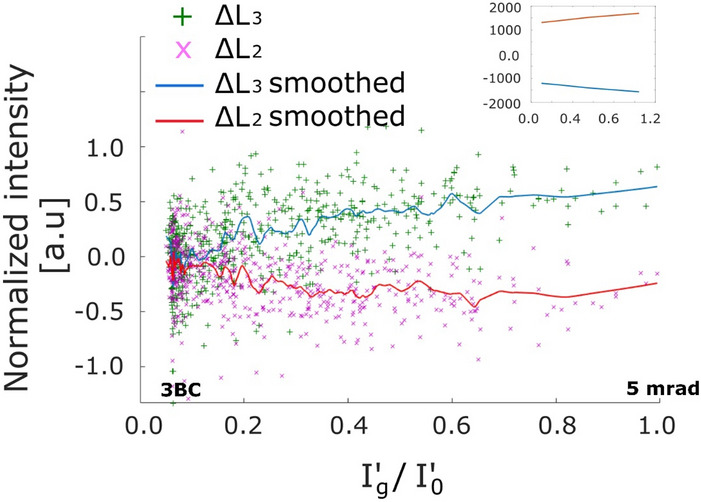
The difference (Δ) signal at L_3_ and L_2_ energy loss edges of Fe plotted against the ${{\varvec{I}}}_{{\varvec{g}}}^{\boldsymbol{^{\prime}}}/{{\varvec{I}}}_{0}^{\boldsymbol{^{\prime}}}$ values. The scatter plots are the experimental values whereas the solid curves are the running average of the experimental data over an interval of 0.05. The inset shows the simulated magnetic signal in the same tilt range as for the experimental data. The axes labels for the inset figure are same as the main figure, with the difference that the values in the experimental plot are normalized to the maximum L_3_ edge intensities in the originally acquired EELS spectra (described in “Methods” section).

To compare the variation of the magnetic signal with a change in crystal tilt in the experimental data to the simulations, we plotted the difference (EMCD signal) at L_3_ and L_2_ energy loss edges of Fe against the ${I}_{g}^{^{\prime}}/{I}_{0}^{^{\prime}}$ intensity with ${I}_{g}^{^{\prime}}/{I}_{0}^{^{\prime}}$ data ordered from the smallest to the largest values as presented in Fig. 6. Looking at the Fig. 3 (c), the value of ${I}_{g}^{^{\prime}}/{I}_{0}^{^{\prime}}$ reduces to 0.1 with approximately 5 mrad tilt from EIP1 towards 3-beam orientation. Thus, the tilt range of ${I}_{g}^{^{\prime}}/{I}_{0}^{^{\prime}}$ axis in Fig. 6 can be considered 5 mrad when ${I}_{g}^{^{\prime}}/{I}_{0}^{^{\prime}}$ ranges from 0.1–1.0 where 1.0 represents the EIP1 orientation and 0.1 corresponds to an orientation close to the 3BC in Fig. 3. The values of ${I}_{g}^{^{\prime}}/{I}_{0}^{^{\prime}}$ less than 0.1 represent a negative tilt from the 3-beam orientation. The inset in Fig. 6 shows the simulated magnetic curves extracted from Fig. 3c in the tilt range determined for the experimental data. There is a close match between the simulated and the experimental magnetic curves. The experimental results confirm the variation of the magnetic signal as a function of crystal tilt observed in the simulations.

**Figure 7:**
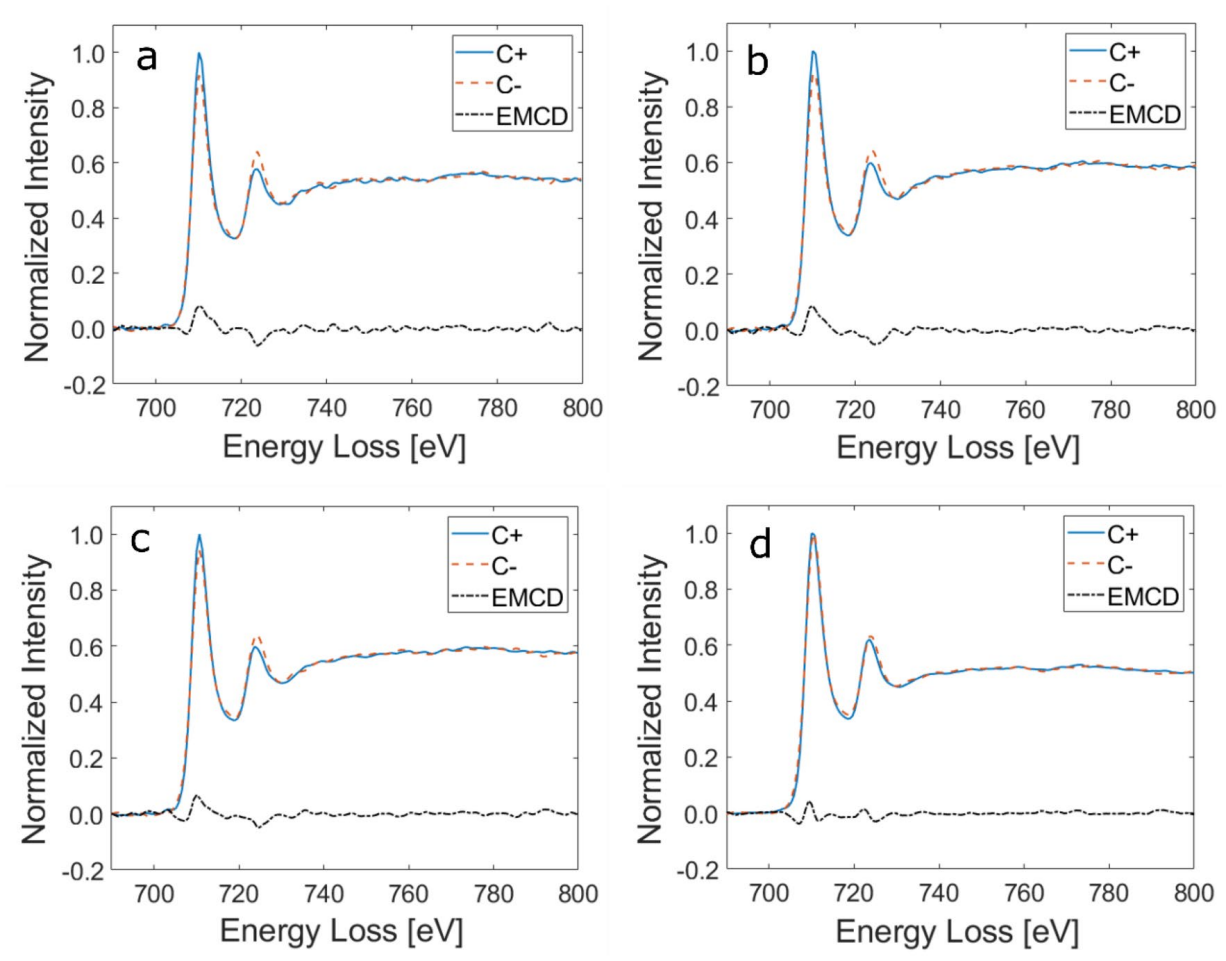
EMCD spectra extracted from the map with (${{\varvec{I}}}_{{\varvec{g}}}^{\boldsymbol{^{\prime}}}/{{\varvec{I}}}_{0}^{\boldsymbol{^{\prime}}}$) orientation intervals **(a)** 0.76–1.0 **(b)** 0.51–0.75 **(c)** 0.26–0.50 **(d)** 0.0–0.25. The total number of spectra summed up for individual intervals are different.

Both the experimental results and the simulations show that the EMCD signal gradually decreases but is relatively stable for a crystal tilt from 2-beam orientation to 3-beam orientation. To investigate the effects of this change in the crystal orientation on the measured magnetic properties, we have extracted the EMCD signals for different crystal tilt intervals. For this purpose, we divided the data into four ${I}_{g}^{^{\prime}}/{I}_{0}^{^{\prime}}$ intervals of 0.76–1.0, 0.50–0.75, 0.26–0.50 and 0.0–0.25 and summed up all the EELS spectra within each interval to obtain single chiral plus (C +) and chiral minus (C−) EELS spectra for each interval. The EELS spectra were background subtracted and the post-edge normalized as described in the Methods section. At this stage of post-processing, an additional difficulty is encountered which arises due to the position of the EMCD apertures at different xy-coordinates on the CCD camera. Focussing the EELS edges for both apertures during the experiment is not trivial and there are still some distortions left in the lower spectral trace. These distortions result in blurring the energy loss edges causing the edges in the lower spectral trace to be a little broader than the upper spectral trace. Here, we adopted the profile matching routine recently used in Ref.^31^ to sharpen the edges in the lower trace keeping the area under the edge same. After profile matching, the difference of the two spectra is taken and reported as EMCD signal in Table 1. The EMCD signals obtained for different ${I}_{g}^{^{\prime}}/{I}_{0}^{^{\prime}}$ intervals are shown in Fig. 7. A clear EMCD signal at both the L_3_ and L_2_ edges can be observed in all the cases. The strength of the EMCD signal at L_3_ edge goes down from 8.3 to 4% for the EIP1 to the close to 3-beam orientation case orientation whereas for the L_2_ edge, it decreases from 6.3 to 3.8% for the same respective orientations.

**Table 1:** The calculated values of m_L_/m_S_ from the EMCD spectra obtained for different intervals of I′_g_/I′_0_.

l′_g_/I′_0_ range	Tilt from 2-beam orientation	Intensity difference at L3	Intensity difference at L2	Calculated m_L_/m_s_
0.76–1.0	2.0–2.5 mrad	0.33 ± 0.01	− 0.22 ± 0.02	0.09 ± 0.03
0.51–0.75	2.5–3.1 mrad	0.33 ± 0.01	− 0.23 ± 0.02	0.08 ± 0.03
0.26–0.50	3.1–4.0 mrad	0.29 ± 0.05	− 0.19 ± 0.01	0.09 ± 0.05
0.0–0.25	4.0–12 mrad	0.04 ± 0.02	− 0.07 ± 0.02	− 0.13 ± 0.06

We have calculated the ratio of the orbital to spin magnetic moment (m_L_/m_S_) of iron using the four EMCD spectra shown above. The intensity difference at L_3_ and L_2_ energy loss edges was calculated by fitting a 3.4 eV window centred at the maxima of the edges and integrating the intensities within the window obtaining m_L_/m_S_ values using the sum rules^32,33^.

1 $$\frac{{m}_{L}}{{m}_{S}}=\frac{2}{3} \frac{{\int }_{L3}\Delta I\left(E\right)dE+{\int }_{L2}\Delta I\left(E\right)dE}{{\int }_{L3}\Delta I\left(E\right)dE-2{\int }_{L2}\Delta I\left(E\right)dE}$$

The m_L_/m_S_ values in Table 1 obtained for the first three cases are similar within the error bars and are also in good agreement with the previously reported values for bcc Fe^5,33–35^.

For the last case, the L_2_ difference signal becomes higher than the L_3_ difference signal, resulting in a negative value of m_L_/m_S_. According to simulations shown in Fig. 3c, the range I′_g_/I′_0_ = 0.0–0.25 corresponds to crystal orientations close to the 3-beam orientation. The bad values of m_L_/m_S_ for this range might be an effect of asymmetry^36^ which becomes dominant for larger tilts. Nevertheless, the EMCD signal strength is relatively stable and the measured m_L_/m_S_ values are not affected within a tilt range of little more than 5 mrad from an exact two-beam orientation towards three-beam orientation.

## Discussion and conclusions

According to the simulations (Fig. 3), the EMCD strength for a 20 nm thick iron sample is maximized at tilts 6–7 mrad from the three-beam orientation (7 mrad is almost precisely the two-beam orientation). The signal strength reduces with a tilt away from the 2-beam condition (2BC) but the reduction is not symmetric in the two tilt directions. The EMCD signal rapidly decreases for a tilt towards EIP2 and becomes 0 within a tilt range of 5 mrad (from 7 to 12 mrad), after which it reverses its sign. On the other hand, the EMCD signal stays relatively stable within a tilt range of 10 mrad for a tilt towards the 3 beam orientation and decreases gradually. Although these results are system and orientation dependent, they can give some qualitative hints, why the EMCD strength was often very weak in actual measurements, in particular L_2_ EMCD signals that were often at the limit of detection or had even disappeared. Apart from measuring a sample with unsuitable thickness (close to extinction distance of the **g** beam) it could be due to setting an orientation with equal intensities of the **0** and **g** beams, tilting too far away from the two-beam orientation. From our results it appears to be safer to first set a three-beam orientation and then quantitatively tilt further towards an exact two-beam orientation (regardless of the relative intensities of the **0** and **g** beam). When the quantitative tilting is not possible or not practical, it is advisable to tilt slowly until the first orientation with equal intensities of **0** and **g** beams is reached and measure there. In an optimal case one reaches the exact two-beam orientation, or a slightly smaller tilt, however, the EMCD strength seems to be relatively robust for tilts few mrad smaller than the exact two-beam orientation. This is confirmed by the experimentally obtained EMCD signals with different crystal orientations lying in between the two and the three beam orientations (Fig. 7).

In conclusions, an experimental methodology has been developed to simultaneously map the EMCD signals and the crystal orientations in a single STEM scan, with the sample tilted to 2-beam condition. The methodology is based on the use of a custom-made quadruple aperture in combination with momentum-resolved EELS acquisition. The setup makes it possible to study the variations in the EMCD signal for slight changes in crystal orientation with high precision. Both the simulations and the experimental results show that the EMCD signal is relatively stable between the 2-beam orientation and 3-beam orientation whereas a small mistilt from 2-beam orientation opposite to 3-beam orientation can significantly reduce the EMCD signal. The results help to understand and improve the detection of EMCD signals in future experiments by setting the appropriate orientation conditions. This setup is only applicable to the simple 2-beam orientation conditions used in most of the EMCD experiments. To carry out such a study for more complex orientation conditions such as zone axis, the EMCD signals and the diffraction patterns can be serially acquired from the same region of the sample. Although it will not be as precise to measure the change of scatter conditions between multiple scans as the setup in this work, it still can give a good estimate about the evolution of EMCD signal for misoriented regions.

## Methods

### Sample fabrication

We used a single crystal bcc Fe film to demonstrate the experiments. The 20 nm thick Fe film was epitaxially grown via direct-current magnetron sputtering in an ultra high vacuum system with a base pressure in the low 10^–9^ mbar and an operationg pressure of 2.7 mbar Ar (99.999 99% purity). Prior to the deposition process, the MgO(001) substrate was annealed for 1 h at 550 ºC, while the deposition temperature was kept at 350 ºC. Finally, an Al_2_O_3_ film (3 nm thick) was deposited using radio-frequency sputtering at room temperature, helping to prevent oxidation of the underlying Fe layer. X-ray diffraction measurements (not shown) were performed with a Philips X-Pert Pro MRD diffractometer (Cu Kα = 1.5418 Å). A Gaussian fit of the Fe rocking curve peak revealed a full width at half maximum of 1º.

### TEM sample preparation

The TEM sample was prepared in a plan-view geometry by mechanical polishing, dimple grinding and Ar- ion milling^37^ from the substrate side until perforation appears. In this geometry, the thickness of the Fe film gradually increases from the edge of the hole to the maximum thickness of 20 nm. In the following EMCD experiments, the measurements were carried out on an area of the sample where the thickness of the magnetic film (Fe) is equal to the 20 nm.

### Experimental conditions

**Figure 8:**
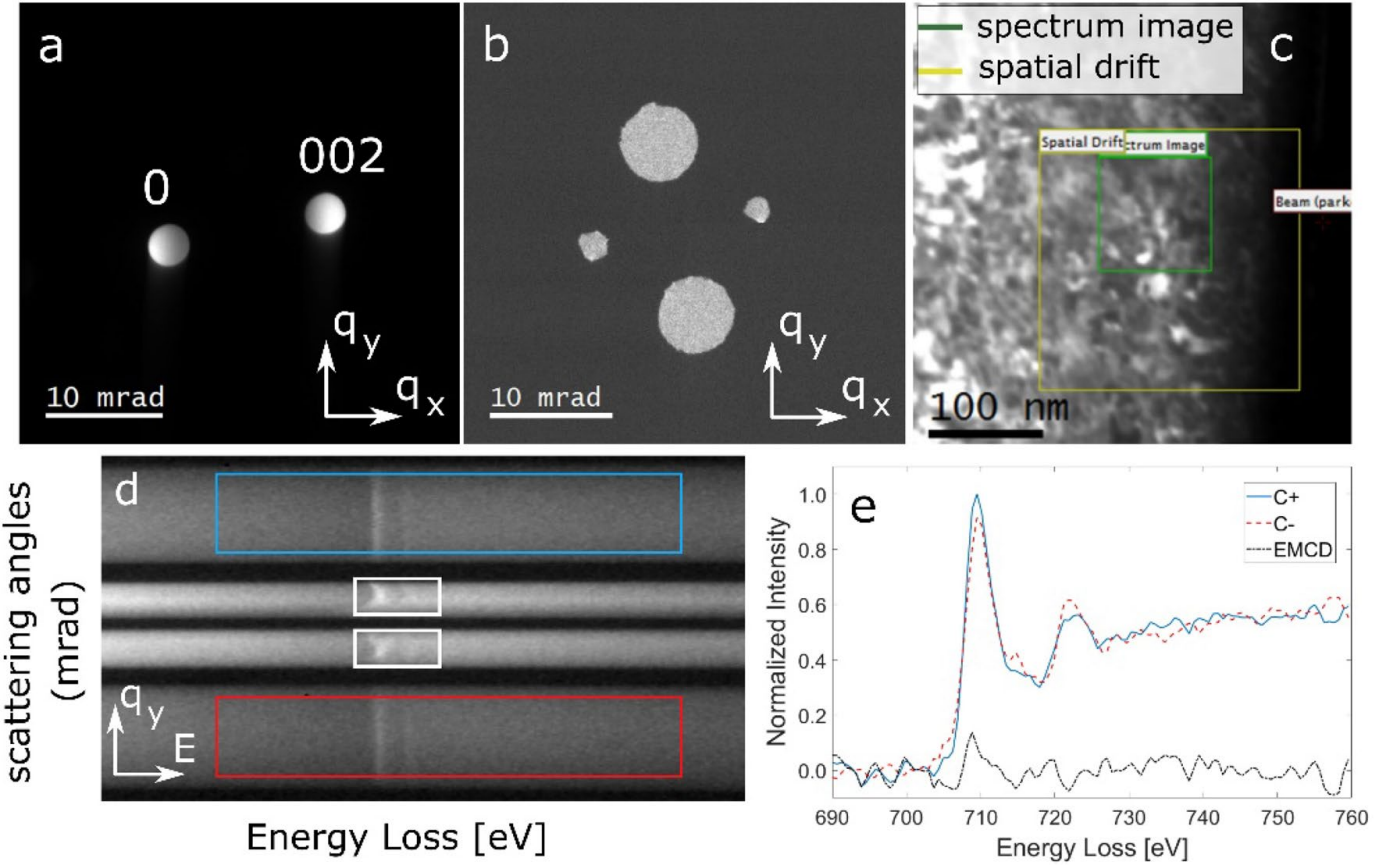
**(a)** Experimentally obtained 2-beam diffraction condition for EMCD experiments with **g** = (002) as diffracted beam. **(b) **An image of the quadruple aperture. **(c) **ADF survey image used to acquire the EMCD-orientation maps with green box showing the measurements area. **(d)** 2D EELS image extracted from one scan point of the acquired 4D data cube. The C + and C- EELS spectra for the EMCD measurements were extracted from the upper and lower traces respectively by integrating the intensities in the marked regions whereas the intensities of **g** and **0** beams were determined by summing up the intensities in the white rectangles marked in the second and third traces respectively. **(e) **The background subtracted and post edge normalized EELS spectra extracted from **d** together with their difference (EMCD) signal.

The experiments were performed on an FEI Tecnai-F30 TEM at an acceleration voltage of 300 kV. The instrument is equipped with a Gatan tridiem spectrometer. To set up the experimental conditions, the TEM sample was tilted away from the [001] zone axis to reach a 2-beam condition by exciting **g** = (002) for Fe as shown in Fig. 8a. A quadruple aperture (QA) was designed with two bigger holes for EMCD measurements and two smaller holes to map the crystal orientation. In the coming text, we will call the bigger aperture holes “EMCD apertures” and the smaller aperture holes “beam apertures” to avoid confusion. The QA aperture was mounted on the spectrometer entrance aperture (SEA) in an orientation that the four holes of the aperture do not overlap along q_y_-axis as shown in Fig. 8b. The TEM sample was rotated so that the positions of the **0** and **g** beams coincide with the positions of the respective beam apertures. Note that despite the rotation of the QA aperture, the EMCD apertures are located at Thales circle positions with respect to the beam apertures. The semi-collection angle for each EMCD aperture is 3 mrad. For the data acquisition, the TEM was operated in microprobe STEM mode and an electron probe with a semi-convergence angle of 1.6 mrad was scanned across a 100 × 100 nm^2^ region of the sample as shown by the green square in Fig. 8c. The step size between two adjacent scan points was set to 4 nm. A 2D EELS image was acquired at each beam position with a dwell time of 5 s producing a 4D datacube. Due to the distinct positions of the four holes of the QA, the momentum transfer along q_y_ is preserved for each aperture resulting in four angle resolved spectroscopic traces being projected simultaneously onto the CCD camera (Fig. 8d). This image contains two conjugate EELS spectral traces (here called C + and C-) produced by the EMCD apertures. At the same time, the inelastic intensities of the **0 (**${I}_{0}^{^{\prime}}$) and **g (**${I}_{g}^{^{\prime}}$) beams are estimated by summing up the edge intensites of the two spectral traces produced by the beam apertures. The image shown in Fig. 8d is extracted from one scan point of the acquired 4D data cube where the edge intensities produced by the **0** and the **g** beam are very close to each other. The C + and C− EELS spectra are extracted from the regions marked by blue and red rectangles on the uppermost and the lowermost spectral traces respectively. The background of the EELS spectra is subtracted by fitting a power law model and extrapolating it under the energy loss edges. After that the post edge of the two spectra is normalized and the EMCD signal is obtained by taking the difference of C + and C− spectra as shown in Fig. 8e. The resulting difference signal shows the EMCD signature with the inverse signs at the L_3_ and somewhat weaker at the L_2_ energy loss edges of Fe.

### Post processing

To produce the maps shown in the results section, MATLAB scripts are written and utilized together with digital micrograph (DMG). In the first step, the energy drift is corrected by aligning the peaks in the 2D EELS images located at different scan points in the 4D dataset. After that, a MATLAB script is used to generate two 3D EELS spectrum images (SI) by extracting the EELS spectra from the uppermost and the lowermost spectral traces in the 2D EELS images at each scan point. The background of each EELS SI is subtracted by fitting and extrapolating a power law background model with a fit window 640–700 eV. The post edge of the EELS spectra situated at each pixel of the background-subtracted SIs is normalized by using a normalization window 750–790 eV. The resulting datasets are here called C + and C− EELS SIs. Afterwards, the EELS spectra at each pixel of C + and C− datasets are divided by the maximum L_3_ edge intensity found at the corresponding pixel of C + dataset to obtain the intensity normalized datasets. The L_3_ and L_2_ difference maps are then produced by fitting an energy window centred at the maxima and calculating the intensity difference between L_3_ and L_2_ edges of the EELS spectra at each corresponding pixel of the intensity normalized C + and C− datasets. Another MATLAB script calculates ${I}_{g}^{^{\prime}}/{I}_{0}^{^{\prime}}$ ratio at each scan point of the 4D dataset by extracting the intensities of the **0** and the **g** beams in the white rectangles marked in Fig. 8d. The ${I}_{g}^{^{\prime}}/{I}_{0}^{^{\prime}}$ map is then generated by putting the values back at the corresponding pixels in a 2D matrix.
